# A refined medium to enhance the antimicrobial activity of postbiotic produced by *Lactiplantibacillus plantarum* RS5

**DOI:** 10.1038/s41598-021-87081-6

**Published:** 2021-04-07

**Authors:** May Foong Ooi, Hooi Ling Foo, Teck Chwen Loh, Rosfarizan Mohamad, Raha Abdul Rahim, Arbakariya Ariff

**Affiliations:** 1 Department of Bioprocess Technology, Faculty of Biotechnology and Biomolecular Sciences, Universiti Putra Malaysia, 43400 UPM Serdang, Selangor Malaysia; 2 Institute of Bioscience, Universiti Putra Malaysia, 43400 UPM Serdang, Selangor Malaysia; 3 Department of Animal Science, Faculty of Agriculture, Universiti Putra Malaysia, 43400 UPM Serdang, Selangor Malaysia; 4 Institute of Tropical Agriculture and Food Security, Universiti Putra Malaysia, 43400 UPM Serdang, Selangor Malaysia; 5 Institute of Tropical Forestry and Forest Products, Universiti Putra Malaysia, 43400 UPM Serdang, Selangor Malaysia; 6 Department of Cell and Molecular Biology, Faculty of Biotechnology and Biomolecular Sciences, Universiti Putra Malaysia, 43400 UPM Serdang, Selangor Malaysia; 7 Office of Vice-Chancellor, Universiti Teknikal Malaysia Melaka, Jalan Hang Tuah Jaya, Durian Tunggal, 76100 Melaka, Malaysia

**Keywords:** Industrial microbiology, Antimicrobials, Applied microbiology, Industrial microbiology, Microbiology

## Abstract

Postbiotic RS5, produced by *Lactiplantibacillus plantarum* RS5, has been identified as a promising alternative feed supplement for various livestock. This study aimed to lower the production cost by enhancing the antimicrobial activity of the postbiotic RS5 by improving the culture density of *L. plantarum* RS5 and reducing the cost of growth medium. A combination of conventional and statistical-based approaches (Fractional Factorial Design and Central Composite Design of Response Surface Methodology) was employed to develop a refined medium for the enhancement of the antimicrobial activity of postbiotic RS5. A refined medium containing 20 g/L of glucose, 27.84 g/L of yeast extract, 5.75 g/L of sodium acetate, 1.12 g/L of Tween 80 and 0.05 g/L of manganese sulphate enhanced the antimicrobial activity of postbiotic RS5 by 108%. The cost of the production medium was reduced by 85% as compared to the commercially available de Man, Rogosa and Sharpe medium that is typically used for *Lactobacillus* cultivation. Hence, the refined medium has made the postbiotic RS5 more feasible and cost-effective to be adopted as a feed supplement for various livestock industries.

## Introduction

*Lactobacillus* is one of the core genera of lactic acid bacteria (LAB). It is broadly defined as a group of Gram-positive, non-sporulating, non-respiring, catalase-negative bacterial species that produce lactic acid as the major end-product from carbohydrate fermentation^1^. It consists of 261 species which are extremely diverse phenotypically and ecologically. The *Lactobacillus* genus has been recently reclassified into 25 new genera based on polyphasic approaches, which includes host-adapted LAB of *Lactobacillus delbrueckii* group, *Paralactobacillus* and 23 novel genera of *Acetilactobacillus, Agrilactobacillus, Amylolactobacillus, Apilactobacillus, Bombilactobacillus, Companilactobacillus, Dellaglioa, Fructilactobacillus, Furfurilactobacillus, Holzapfelia, Lacticaseibacillus, Lactiplantibacillus, Latilactobacillus, Lapidilactobacillus, Lentilactobacillus, Levilactobacillus, Ligilactobacillus, Limosilactobacillus, Liquorilactobacillus, Loigolactobacilus, Paucilactobacillus, Schleiferilactobacillus* and *Secundilactobacillus*^2^. Recently, the name *Lactiplantibacillus plantarum* was suggested for the plantarum-group lactobacilli^2^. This genus is generally associated with carbohydrate-rich environments, such as food, plants and mucosal surfaces of animals and humans^3–5^. Many strains of *Lactiplantibacillus plantarum* (formerly was known as *Lactobacillus plantarum*) have been reported to produce postbiotics that can be employed as natural bio-preservatives for dairy products^6,7^. It has also been suggested as an alternative feed-additive to replace in-feed antimicrobial growth promoter (AGP) to enhance the general health and growth performance of various livestock^8,9^.

Postbiotics are defined as non-viable soluble bioactive metabolites produced by probiotic LAB, which mediate beneficial probiotic effects. The composition of soluble bioactive components of postbiotics can vary under different fermentation conditions, such as growth medium and physical parameters applied for the fermentation process. Organic acids, bacteriocins, ethanol, fatty acids, diacetyl, acetaldehyde and other low molecular mass compounds^10,11^ have been reported to be the bioactive components of postbiotics. Postbiotics are also known as metabiotics, biogenics, metabolites, fermented cell-free supernatants, soluble factors, or metabolic byproducts secreted by live bacteria or released after bacterial lysis. Postbiotics have been demonstrated to offer physiological benefits to the animal host by improving the mucosal gut barrier integrity and reducing pathogen-induced inflammation^12–14^.

Several *L. plantarum* have been isolated from Malaysia foods, such as *Tapai Ubi,* fermented tapioca^5,15^, *Budu,* fermented fish sauce^16^, *Tempoyak*, fermented durian pulp^15,17^ and steamed fish^4^. The postbiotics produced by strains of *L. plantarum* (*L. plantarum* RS5, *L. plantarum* I-UL4, *L. plantarum* RI11 and *L. plantarum* RG14) exhibited broad inhibitory activity against *Pediococcus acidilactici*, *Listeria monocytogenes*, *Salmonella enterica, Escherichia coli* and *Vancomycin resistant enterococci*^18,19^. They also exerted selective cytotoxic and antiproliferative effects and induction of apoptosis against cancer cells in a strain-specific and cancer cell type-specific manner whilst sparing normal cells. Hence, the postbiotics that produced by strains of *L. plantarum* possess potential for use as functional supplements and as adjunctive treatments for cancer^20,21^. The postbiotics produced by these *L. plantarum* strains has been verified in numerous studies as a promising in-feed growth promoter for laying hens^22^, broiler chickens^8,23^, postweaning piglets^24^ and postweaning lambs^9^. Prudent use of AGP in animal feeding results in antibiotic resistance that imparts adverse effects on the environment, animal and human health and hence alternatives, such as postbiotics, are being actively sought^25,26^.

Postbiotic RS5, produced by *L. plantarum* RS5, has been shown to improve the gut health and overall growth performance of the laying hens, broilers and postweaning piglets^22–24^. Bacteriocin is one of the crucial components that contribute predominantly to the broad antimicrobial activity to promote the gut health and overall growth performance of the tested animals. However, the production of bacteriocin is not naturally optimised^27^. Hence, this has driven the current research to enhance the antimicrobial activity of postbiotic RS5.

Previous research findings have shown that medium composition can have a strong effect on bacteriocin production and the growth of LAB producer cells^28–32^. de Man, Rogosa and Sharpe Medium (MRS) is one of the commercially available selective media, which is commonly employed as a reference in growth medium optimisation studies^28,30^. Frequently, the medium composition is optimised via either conventional methods, statistical methods, or a combination of both conventional and statistical methods^28,33–35^. The conventional method involves changing one independent variable at a time while maintaining other variables at a fixed level. This method is simple to implement and mainly assists in the selection of significant factors affecting the yield of desired products. However, the interaction effects of various physicochemical parameters will not be elucidated accordingly^30,34^.

In comparison, a more comprehensive statistical-based optimisation design can facilitate the understanding of interaction effects between the studied parameters^35^. Fractional factorial design [FFD] and response surface methodology (RSM) are experimental strategies that gather the statistical techniques for designing experiments, evaluating the effects of independent variables and searching for the optimum conditions of variables to achieve a desirable goal^36,37^. This method has been successfully applied for the optimisation of metabolite production by microorganisms^34,38,39^, conditions of enzymatic hydrolysis^40^, amino acid productions^40–44^ and parameters for food preservation and fermentation processes^45^. This study aimed to enhance the antimicrobial activity of the postbiotic RS5 by improving the culture density of *L. plantarum* RS5 and reduce the cost of the growth medium. Therefore, a combination of conventional and statistical-based approaches (FFD and Central Composite Design of RSM) was employed to develop a refined medium for the enhancement of the antimicrobial activity of postbiotic RS5.

## Results

### Conventional refinement of antimicrobial activity of postbiotic RS5

#### Optimum concentration of yeast extract nitrogen source

**Figure 1:**
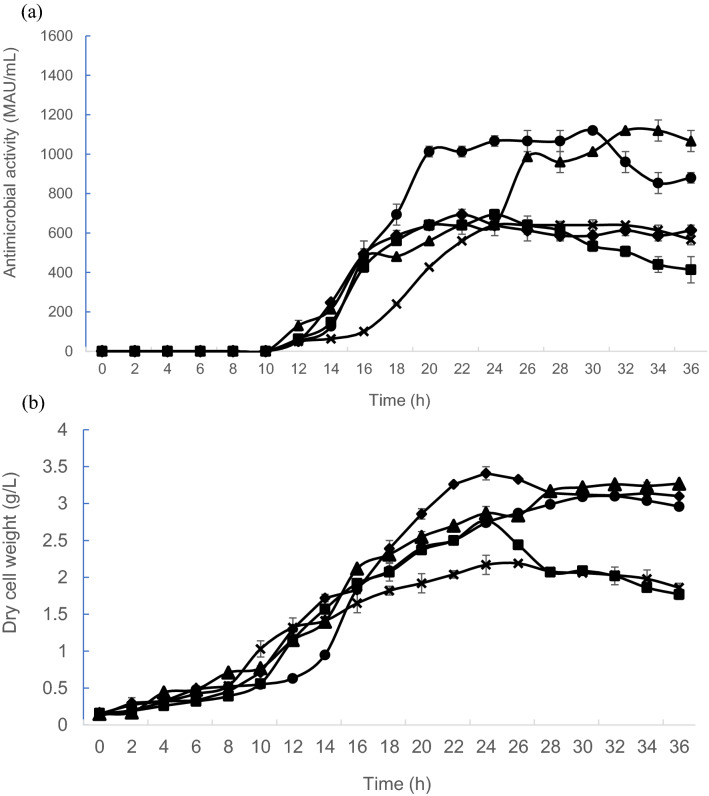
Effects of different yeast extract concentrations on antimicrobial activity of postbiotic RS5 and biomass produced by *L. plantarum* RS5. (**a**) Antimicrobial activity profiles of postbiotic RS5 and (**b**) growth profile of *L. plantarum* RS5 in control MRS medium (x) and growth medium containing 11.89 g/L (85.49 mM N) (filled square), 27.84 g/L (200.18 mM N) (filled circle), 36.20 g/L (260.28 mM N) (filled triangle) and 44.55 g/L of yeast extract (320.32 mM N) (filled rhombus). Values presented are mean ± SEM, n = 3. Vertical bars represent SEM.

The total nitrogen content (N) of KAT yeast extract was previously determined using Kjeltec 2400 (Foss, UK) equipment. Figure 1a shows the antimicrobial activity of postbiotic RS5 was initially detected at 12 h of incubation time for all yeast extract concentrations used in this experiment [85.49 mM (11.89 g/L), 200.18 mM (27.84 g/L), 260.28 mM (36.20 g/L), and 320.32 mM (44.55 g/L) N]. However, the maximum antimicrobial activity of 1120 MAU/mL was achieved at 30 h and at 32 h of incubation time in growth medium containing 27.84 g/L and 36.20 g/L of yeast extract respectively, in comparison to control MRS medium and other yeast extract concentrations. Interestingly, biphasic exponential production was noted between 10 to 14 h and 18 to 22 h of incubation for 27.84 g/L (200.18 mM N) of yeast extract. At 36.20 g/L (260.28 mM N) of yeast extract, the biphasic exponential production of antimicrobial activity was noted between 10 to 18 h and between 24 to 26 h of incubation.

Figure 1b shows the effect of different concentrations of yeast extract concentrations on the growth of *L. plantarum* RS5. As for the 27.84 g/L of yeast extract, the maximum biomass of *L. plantarum* RS5 (3.10 g/L) was achieved at 32 h of incubations. The biomass was increased to 3.26 g/L (32 h) when the yeast extract concentration was increased to 36.20 g/L. The significantly highest (p < 0.05) biomass of 3.41 g/L (24 h) was attained in the growth medium containing 44.55 g/L of yeast extract. However, significantly lower (p < 0.05) antimicrobial activity was detected in the growth medium containing 44.55 g/L of yeast extract, in comparison to the growth medium containing 27.84 g/L and 36.20 g/L of yeast extract, respectively.

Table 1 shows the kinetic parameters of *L. plantarum* RS5 when cultivated in growth medium containing different concentrations of yeast extract. The highest productivity of antimicrobial activity of 37.33 MAU/mL h was achieved with 27.84 g/L of yeast extract as a comparison to the MRS medium that contributed to the lowest productivity of 26.67 MAU/mL h. Furthermore, the highest specific growth rate (µ) of *L. plantarum* RS5 (0.24 h^−1^) was achieved with 27.84 g/L of yeast extract, indicating that the 27.84 g/L (200.18 mM N) was the optimum concentration of yeast extract for the growth of *L. plantarum* RS5 to produce the highest antimicrobial activity in postbiotic RS5.

**Table 1:** Effects of different concentrations of yeast extract on the growth and antimicrobial activity of postbiotic RS5 produced by *L. plantarum* RS5.

Kinetic parameters*	Control MRS medium	Yeast extract concentration (g/L)			
		11.89	27.84	36.20	44.55
P_max_ (MAU/mL)**	640.00^b^ ± 0.00	640.00^b^ ± 0.00	1120^a^ ± 0.00	1120.00^a^ ± 0.00	693.33^b^ ± 26.67
t (h)	24	20	30	32	22
Pr (MAU/mL h)	26.67	32	37.33	35	31.52
X_max_ (g/L)**	2.19^d^ ± 0.02	2.77^c^ ± 0.02	3.10^b^ ± 0.12	3.26^b^ ± 0.02	3.41^a^ ± 0.01
µ (h^−1^)	0.20	0.23	0.24	0.23	0.15
t_x_ (h)	26	24	32	32	20

*P_max_ (MAU/mL)—maximum antimicrobial activity, t (h)—cultivation time of maximum antimicrobial activity, Pr (MAU/mL h)—productivity of antimicrobial activity, X_max_ (g/L)—maximum biomass, µ (h^−1^)—specific growth rate, t_x_ (h)—cultivation time of maximum biomass.

**Values for P_max_ (MAU/mL) and X_max_ (g/L) are mean ± standard error of the mean (SEM), n = 3.

Mean ± SEM within the same row that does not share a similar superscript are significantly different (p < 0.05).

#### Effect of different carbon sources

**Figure 2:**
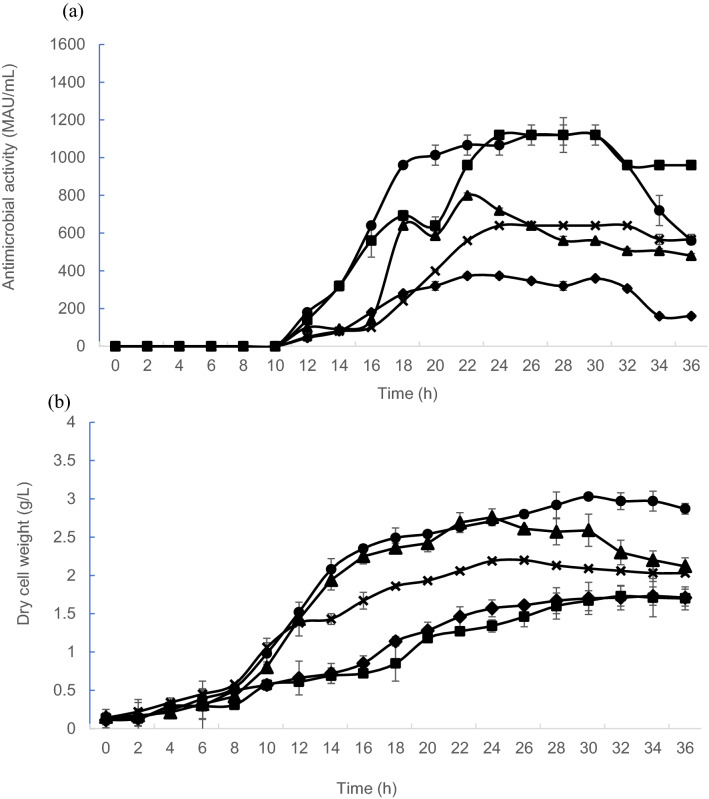
Effects of different carbon sources on antimicrobial activity of postbiotic RS5 and biomass produced by *L. plantarum* RS5. (**a**) Antimicrobial activity profiles of postbiotic RS5 and (**b**) growth of *L. plantarum* RS5 in control MRS medium (x) and growth medium containing glucose (filled circle), lactose (filled square), fructose (filled rhombus) and sucrose (filled triangle). Values presented are mean ± SEM, n = 3. Vertical bars represent SEM.

*Lactiplantibacillus plantarum* RS5 produced different levels of antimicrobial activities when cultivated in growth medium containing different carbon sources. The production of antimicrobial activity was initiated after 10 h of incubation for all the tested carbon sources. The profile of antimicrobial activities (Fig. 2a) showed that significantly higher (p < 0.05) antimicrobial activities were achieved with the growth medium containing glucose or lactose as the carbon source in comparison to fructose and sucrose as carbon source throughout the incubation period. The highest antimicrobial activity of 1120 MAU/mL was observed from 24 to 30 h of incubation when lactose or glucose was used as the carbon source. The highest antimicrobial activity achieved using sucrose was only 800 MAU/mL, which was 29% lower than the highest antimicrobial activity that was achieved with glucose and lactose. However, the 800 MAU/mL of antimicrobial activity was 20% higher than the antimicrobial activity detected when MRS medium was used.

The exponential growth of *L. plantarum* RS5 in growth medium containing sucrose was noted between 8 and 16 h, which was similar to the growth profile that observed in growth medium containing glucose as carbon source (Fig. 2b). Comparable biomass was detected when *L. plantarum* RS5 was grown in medium containing glucose or sucrose as carbon source until 24 h of incubation. The highest biomass (3.03 g/L) of *L. plantarum* RS5 was achieved in growth medium containing glucose at 30 h of incubation. Surprisingly, the lowest biomass was attained when *L. plantarum* RS5 was grown in medium containing lactose as a carbon source, followed by fructose and MRS medium.

The kinetic analysis (Table 2) showed that the highest specific growth rate of *L. plantarum* RS5 was achieved with glucose (0.32 h^−1^), followed by sucrose (0.24 h^−1^). Although *L. plantarum* RS5 produced the highest antimicrobial activity in lactose containing growth medium, the lowest specific growth rate (0.06 h^−1^) and biomass (1.73 g/L) were detected when lactose was supplemented in the growth medium as carbon source. The same level of the highest antimicrobial activity was produced at 26–30 h of incubation when *L. plantarum* RS5 was grown in medium containing either glucose or lactose as carbon source. In addition, the highest biomass of *L. plantarum* RS5 was also achieved in growth medium containing glucose. Thus, glucose was selected as the best carbon source for the subsequent experiments, since the cost of glucose was lower in comparison to lactose to produce *L. plantarum* RS5 biomass and the antimicrobial activity of postbiotic RS5.

**Table 2:** Effects of carbon sources on the growth and antimicrobial activity of postbiotic RS5 produced by *L. plantarum* RS5.

Kinetic parameters*	Control MRS medium	Carbon sources			
		Glucose	Lactose	Fructose	Sucrose
P_max_ (MAU/mL)**	640.00^c^ ± 0.00	1120.00^a^ ± 0.00	1120.00^a^ ± 46.19	373.33^d^ ± 13.33	800.00^b^ ± 0.00
t (h)	24	26	24	22	22
Pr (MAU/mL h)	26.67	43.08	46.67	16.97	36.36
X_max_ (g/L)**	2.20^b^ ± 0.03	3.03^a^ ± 0.03	1.73^c^ ± 0.13	1.73^c^ ± 0.12	2.76^b^ ± 0.09
µ (h^−1^)	0.20	0.32	0.06	0.15	0.24
t_x_ (h)	26	30	32	34	24

*P_max_ (MAU/mL)—maximum antimicrobial activity, t (h)—cultivation time of maximum antimicrobial activity, Pr (MAU/mL h)—productivity of antimicrobial activity, X_max_ (g/L)—maximum biomass, µ (h^−1^)—specific growth rate, t_x_ (h)—cultivation time of maximum biomass.

**Values for P_max_ (MAU/mL) and X_max_ (g/L) are mean ± standard error of the mean (SEM), n = 3.

Mean ± SEM within the same row that does not share a similar superscript are significantly different (p < 0.05).

#### Effect of glucose concentrations

**Figure 3:**
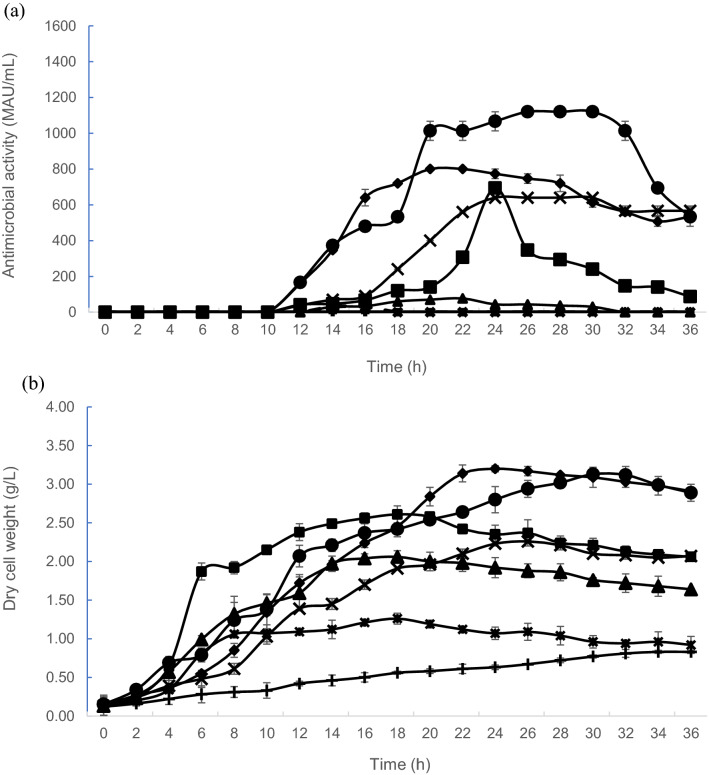
Effects of different carbon glucose concentrations on antimicrobial activity of postbiotic RS5 and biomass produced by *L. plantarum* RS5. (**a**) Antimicrobial activity profiles of postbiotic RS5 and (**b**) growth of *L. plantarum* RS5 in control MRS medium (x) and growth medium containing 0 g/L (+); 5 g/L (*); 10 g/L (filled triangle); 15 g/L (filled square); 20 g/L (filled circle) and 40 g/L (filled rhombus) of glucose. Values presented are mean ± SEM, n = 3. Vertical bars represent SEM.

Different levels of antimicrobial activities (Fig. 3a) were observed when *L. plantarum* RS5 was cultivated in growth medium containing different glucose concentrations. No antimicrobial activity was detected when glucose was not supplemented in the growth medium. An obvious increment of antimicrobial activity was observed when *L. plantarum* RS5 was cultivated in growth medium containing 20 g/L of glucose, whereby 1013.33 MAU/mL to 1120 MAU/mL of antimicrobial activity was achieved between 20 and 32 h of incubation. Surprisingly, a twofold reduction of antimicrobial activity (640–800 MAU/mL) was detected at 18–30 h of incubation, when the glucose concentration was increased to 40 g/L. Similarly, the antimicrobial activity of postbiotic RS5 was significantly lower (p < 0.05) when glucose was supplemented at a concentration that lower than 20 g/L, with 30 MAU/mL at 14 h of incubation, 76.67 MAU/mL at 22 h of incubation and 693.33 MAU/mL at 24 h was produced when 5 g/L, 10 g/L and 15 g/L of glucose was used, respectively.

The growth profile (Fig. 3b) showed that *L. plantarum* RS5 produced different levels of biomass when cultivated in a growth medium containing different glucose concentrations. Higher glucose concentrations (15 g/L, 20 g/L and 40 g/L) promoted higher biomass formation throughout the cultivation period. Comparable biomass was attained when *L. plantarum* RS5 was cultivated in the growth medium containing 20 or 40 g/L of glucose at 28–32 h of incubation. However, the highest biomass was achieved at 22 h of incubation when *L. plantarum* RS5 was grown in a growth medium containing 40 g/l glucose.

Similarly, different specific growth rates were detected when of *L. plantarum* RS5 was cultivated in growth medium containing different levels of glucose (Table 3). The highest specific growth rate of *L. plantarum* RS5 was achieved with growth medium containing 20 g/L of glucose.

**Table 3:** Effect of glucose concentrations on the growth and antimicrobial activity of postbiotic RS5 produced by *L. plantarum* RS5.

Kinetic parameters*	Control MRS medium	Glucose concentration (g/L)					
		0	5	10	15	20	40
P_max_ (MAU/mL)**	640.00^c^ ± 0.00	0.00^f^ ± 0.00	30.00^e^ ± 0.00	76.67^d^ ± 3.33	693.33^c^ ± 26.67	1120.00^a^ ± 0.00	800.00^b^ ± 0.00
t (h)	24	0	14	22	24	26	20
Pr (MAU/mL h)	26.67	0.00	2.14	3.49	28.89	43.08	40.00
X_max_ (g/L)**	2.26^e^ ± 0.01	0.83^g^ ± 0.12	1.26^f^ ± 0.07	2.06^c^ ± 0.08	2.61^d^ ± 0.11	3.20^a^ ± 0.04	3.13^b^ ± 0.04
µ (h^−1^)	0.20	0.05	0.08	0.11	0.21	0.31	0.28
t_x_ (h)	26	34	18	18	18	30	24

*P_max_ (MAU/mL)—maximum antimicrobial activity, t (h)—cultivation time of maximum antimicrobial activity, Pr (MAU/mL h)—productivity of antimicrobial activity, X_max_ (g/L) —maximum biomass, µ (h^−1^)—specific growth rate, t_x_ (h)—cultivation time of maximum biomass.

**Values for P_max_ (MAU/mL) and X_max_ (g/L) are mean ± standard error of the mean (SEM), n = 3.

Mean ± SEM within the same row that does not share a similar superscript are significantly different (p < 0.05).

### Statistical refinement of antimicrobial activity of postbiotic RS5

#### Selection of significant ionic minerals and non-ionic surfactant by FFD

Table 4 shows the experimental design, antimicrobial activities, biomass production, lactate production and the corresponding pH of postbiotic RS5 produced by *L. plantarum* RS5. The growth media comprised different combinations of ionic minerals and a non-ionic surfactant in addition to 20 g/L of glucose and 27.84 g/L of yeast extract as carbon and nitrogen source. The antimicrobial activities and biomass varied markedly from 0 to 1280 MAU/mL and 0.94 g/L to 3.35 g/L, respectively. The results showed that the highest antimicrobial activity (1280 MAU/mL) was achieved together with the highest biomass (3.35 g/L) and lactate (7.07 g/L) production (run 7).

**Table 4:** FFD matrix with coded and real values of ionic minerals, non-ionic surfactant and their corresponding effects on the antimicrobial activity, lactate concentration and pH of postbiotic RS5, and biomass of *L. plantarum* RS5.

Run	Ionic minerals and non-ionic surfactant (g/L)*						Antimicrobial activity (MAU/mL)**	Biomass (g/L)**	Lactate (g/L)**	Final pH**
	X_1_	X_2_	X_3_	X_4_	X_5_	X_6_				
1	5.00 (+ 1)	0.00 (− 1)	2.00 (+ 1)	0.00 (− 1)	0.00 (− 1)	0.00 (− 1)	0.00^j^ ± 0.00	1.08^fg^ ± 0.02	2.28^hi^ ± 0.09	4.18^j^ ± 0.06
2	5.00 (− 1)	0.00 (− 1)	0.00 (− 1)	1.00 (+ 1)	0.20 (+ 1)	0.04 (+ 1)	560.00^e^ ± 0.00	2.24^bc^ ± 0.06	5.60^b^ ± 0.09	3.88^ h^ ± 0.01
3	0.00 (− 1)	0.00 (− 1)	2.00 (+ 1)	0.00 (− 1)	0.20 (+ 1)	0.00 (− 1)	0.00^j^ ± 0.00	1.07^g^ ± 0.02	2.24^hij^ ± 0.04	4.19^j^ ± 0.01
4	5.00 (+ 1)	2.00 (+ 1)	2.00 (+ 1)	1.00 (+ 1)	0.20 (+ 1)	0.04 (+ 1)	1120.00^b^ ± 0.00	3.21^a^ ± 0.02	6.97^a^ ± 0.05	3.77^a^ ± 0.01
5	0.00 (− 1)	2.00 (+ 1)	2.00 (+ 1)	1.00 (+ 1)	0.20 (+ 1)	0.00 (− 1)	360.00^f^ ± 0.00	1.90^d^ ± 0.04	3.22^e^ ± 0.05	4.15^d^ ± 0.02
6	0.00 (− 1)	0.00 (− 1)	2.00 (+ 1)	1.00 (+ 1)	0.00 (− 1)	0.00 (− 1)	146.67^gh^ ± 17.63	1.21^fg^ ± 0.07	2.91^ef^ ± 0.05	4.19^ h^ ± 0.05
7	5.00 (+ 1)	0.00 (− 1)	2.00 (+ 1)	1.00 (+ 1)	0.00 (− 1)	0.04 (− 1)	1280.00^a^ ± 0.00	3.35^a^ ± 0.03	7.07^a^ ± 0.02	3.73^b^ ± 0.02
8	0.00 (− 1)	0.00 (− 1)	2.00 (+ 1)	0.00 (− 1)	0.00 (− 1)	0.04 (+ 1)	40.00^ij^ ± 0.00	1.01^g^ ± 0.01	2.15^hij^ ± 0.12	4.31^b^ ± 0.01
9	5.00 (+ 1)	2.00 (+ 1)	0.00 (− 1)	1.00 (+ 1)	0.20 (+ 1)	0.00 (− 1)	333.33^f^ ± 26.67	1.88^d^ ± 0.06	3.09^e^ ± 0.04	4.28^f^ ± 0.01
10	0.00 (− 1)	0.00 (− 1)	0.00 (− 1)	0.00 (− 1)	0.00 (− 1)	0.00 (− 1)	40.00^ij^ ± 0.00	1.16^fg^ ± 0.03	2.91^ef^ ± 0.05	4.22^b^ ± 0.05
11	5.00 (+ 1)	0.00 (− 1)	0.00 (− 1)	0.00 (− 1)	0.20 (+ 1)	0.00 (− 1)	0.00^j^ ± 0.00	1.04^g^ ± 0.10	2.24^hij^ ± 0.03	4.19^e^ ± 0.02
12	0.00 (− 1)	2.00 (+ 1)	2.00 (+ 1)	0.00 (− 1)	0.20 (+ 1)	0.04 (+ 1)	80.00^hij^ ± 0.00	1.11^fg^ ± 0.01	2.30^hi^ ± 0.04	4.10^b^ ± 0.01
13	5.00 (+ 1)	2.00 (+ 1)	2.00(+ 1)	1.00 (+ 1)	0.00 (− 1)	0.00 (− 1)	333.33^f^ ± 26.67	1.91^d^ ± 0.08	3.16^e^ ± 0.08	4.24^b^ ± 0.02
14	5.00 (+ 1)	2.00 (+ 1)	0.00 (− 1)	1.00 (+ 1)	0.00 (− 1)	0.04 (− 1)	746.67^c^ ± 26.67	2.43^b^ ± 0.06	5.73^b^ ± 0.02	3.78^ h^ ± 0.01
15	0.00 (− 1)	0.00 (− 1)	2.00 (+ 1)	1.00 (+ 1)	0.20 (+ 1)	0.04 (− 1)	560.00^e^ ± 0.00	2.22^bc^ ± 0.05	5.87^b^ ± 0.11	3.75^b^ ± 0.01
16	0.00 (− 1)	2.00 (+ 1)	0.00 (− 1)	0.00 (− 1)	0.20 (+ 1)	0.00 (− 1)	0.00^j^ ± 0.00	1.06^g^ ± 0.02	2.36^hi^ ± 0.13	4.14^j^ ± 0.03
17	5.00 (+ 1)	2.00 (+ 1)	2.00 (+ 1)	0.00 (− 1)	0.20 (+ 1)	0.00 (− 1)	0.00^j^ ± 0.00	1.08^fg^ ± 0.05	2.42^ghi^ ± 0.06	4.05^ h^ ± 0.04
18	5.00 (+ 1)	0.00 (− 1)	0.00 (− 1)	1.00 (+ 1)	0.00 (− 1)	0.00 (− 1)	280.00^f^ ± 0.00	1.86^d^ ± 0.07	2.84^efg^ ± 0.08	4.07^c^ ± 0.06
19	5.00 (+ 1)	0.00 (− 1)	2.00 (+ 1)	1.00 (+ 1)	0.20 (+ 1)	0.00 (− 1)	173.33^g^ 40.55	1.54^e^ ± 0.06	2.54^fgh^ ± 0.07	4.05^a^ ± 0.04
20	0.00 (− 1)	0.00 (− 1)	0.00 (− 1)	1.00 (+ 1)	0.20(+ 1)	0.00 (− 1)	153.33^gh^ ± 24.01	1.35^ef^ ± 0.03	3.14^e^ ± 0.09	4.27^b^ ± 0.03
21	0.00 (− 1)	2.00 (+ 1)	2.00 (+ 1)	0.00 (− 1)	0.00(− 1)	0.00 (− 1)	0.00^j^ ± 0.00	1.02^g^ ± 0.06	2.08^ l^ ± 0.02	4.32^ h^ ± 0.03
22	0.00 (− 1)	2.00 (+ 1)	0.00 (− 1)	1.00 (+ 1)	0.00 (− 1)	0.00 (− 1)	333.33^f^ ± 35.28	2.01^cd^ ± 0.06	4.95^cd^ ± 0.04	3.88^g^ ± 0.00
23	0.00 (− 1)	0.00 (− 1)	0.00 (− 1)	0.00 (− 1)	0.20 (+ 1)	0.04 (+ 1)	95.00^ghi^ ± 2.89	0.94^g^ ± 0.04	1.80^j^ ± 0.11	4.39^g^ ± 0.01
24	0.00 (− 1)	0.00 (− 1)	0.00 (− 1)	1.00 (+ 1)	0.00(− 1)	0.04 (+ 1)	640.00^de^ ± 0.00	2.34^b^ ± 0.04	5.06^c^ ± 0.04	3.79^g^ ± 0.01
25	5.00 (+ 1)	2.00 (+ 1)	2.00 (+ 1)	0.00 (− 1)	0.00 (− 1)	0.04 (+ 1)	76.67^hij^ ± 6.67	1.06^g^ ± 0.04	2.17^hij^ ± 0.09	4.21^d^ ± 0.01
26	0.00 (− 1)	2.00 (+ 1)	2.00 (+ 1)	1.00 (+ 1)	0.00 (− 1)	0.04 (+ 1)	666.67^cd^ ± 26.67	2.38^b^ ± 0.02	5.60^b^ ± 0.25	3.75^g^ ± 0.01
27	5.00 (+ 1)	2.00 (+ 1)	0.00 (− 1)	0.00 (− 1)	0.20 (+ 1)	0.04 (+ 1)	93.33^ghi^ ± 0.00	1.22^fg^ ± 0.04	3.04^e^ ± 0.04	4.26^b^ ± 0.02
28	5.00 (+ 1)	2.00 (+ 1)	0.00 (− 1)	0.00 (− 1)	0.00 (− 1)	0.00 (− 1)	0.00^j^ ± 0.00	1.04^g^ ± 0.02	1.98^ij^ ± 0.06	4.33^b^ ± 0.02
29	0.00 (− 1)	2.00 (+ 1)	0.00 (− 1)	0.00 (− 1)	0.00(− 1)	0.04 (+ 1)	98.33^ghi^ ± 13.02	1.13^fg^ ± 0.04	2.29^hi^ ± 0.08	4.23^e^ ± 0.02
30	5.00 (+ 1)	0.00 (− 1)	2.00 (+ 1)	0.00 (− 1)	0.20(+ 1)	0.04 (+ 1)	40.00^ij^ ± 0.00	1.09^fg^ ± 0.08	2.02^ij^ ± 0.06	4.31^b^ ± 0.02
31	5.00 (− 1)	0.00 (− 1)	0.00 (− 1)	0.00 (− 1)	0.00 (− 1)	0.04 (+ 1)	35.00^ij^ ± 0.00	1.06^g^ ± 0.02	2.19^hij^ ± 0.03	4.21^e^ ± 0.01
32	0.00 (− 1)	2.00 (+ 1)	0.00 (− 1)	1.00 (+ 1)	0.20 (+ 1)	0.04 (+ 1)	560.00^e^ ± 0.00	2.25^bc^ ± 0.05	4.54^d^ ± 0.11	3.92^f^ ± 0.01

*X_1_: Sodium acetate; X_2_: Di-potassium hydrogen phosphate; X_3_: Di-ammonium hydrogen citrate; X_4_: Tween 80; X_5_: Magnesium sulphate heptahydrate; X_6_: Manganese sulphate tetrahydrate. Coded values of medium components are indicated in parentheses.

**Values presented are mean ± standard error of the mean (SEM), n = 3. Mean ± SEM within the same column that does not share a similar superscript are significantly different (p < 0.05).

The interaction effects of media component on antimicrobial activity were evaluated by ANOVA (Table 5). From the ANOVA table, the linear effect of Tween 80 (X4) and manganese sulphate (X6), and the interaction between sodium acetate (X1) and Tween 80 (X4), and between Tween 80 (X4) and manganese sulphate (X6) affected the antimicrobial activity strongly at a p-value less than 0.05. However, the other mineral components did not affect the antimicrobial activity significantly (p > 0.05). The following equation 1 is the model of the desired response (Y_MAU/mL_) and regression coefficients:

1 $$\sqrt{(\text{Y}+12.80)}=6.29 +8.79 {\text{X}}_{4 }+123.30 {\text{X}}_{6 }+0.84 \, {\text{X}}_{1 }{\text{X}}_{4}+155. 44 {\text{X}}_{4}{{\text{X}}}_{6}$$

**Table 5:** ANOVA analyses for FFD models of ionic minerals and non-ionic surfactant on the antimicrobial activity of postbiotic RS5 produced by *L. plantarum* RS5.

Ionic minerals and non-ionic surfactant*	Sum of squares	df	Mean square	F value	Prob > F**
Model	2647.17	13	203.63	33.99	< 0.0001
X_1_	10.79	1	10.79	1.80	0.20
X_2_	19.71	1	19.71	3.29	0.09
X_3_	1.71	1	1.71	0.29	0.60
X_4_	1920.53	1	1920.53	320.63	< 0.0001
X_5_	4.85	1	4.85	0.81	0.38
X_6_	517.20	1	517.20	86.34	< 0.0001
X_1_X_3_	13.67	1	13.67	2.28	0.15
X_1_X_4_	35.55	1	35.55	5.93	0.03
X_1_X_5_	0.48	1	0.48	0.08	0.78
X_3_X_4_	17.72	1	17.72	2.96	0.10
X_3_X_5_	1.70	1	1.70	0.28	0.60
X_4_X_6_	77.31	1	77.31	12.91	0.01
X_1_X_3_X_5_	25.92	1	25.92	4.33	0.07
R-squared	0.9544				
Adjusted R-squared	0.9256				
Adequate precision	17.36				

*X_1_: Sodium acetate; X_2_: Di-potassium hydrogen phosphate; X_3_: Di-ammonium hydrogen citrate; X_4_: Tween 80; X_5_: Magnesium sulphate heptahydrate; X_6_: Manganese sulphate tetrahydrate.

**Value of “Prob > F” less than 0.05 indicate model terms are significant.

whereby Y = antimicrobial activity; X_4_ = tween 80; X_6_ = manganese sulphate tetrahydrate; X_1_ X_4_ = interaction of sodium acetate and tween 80; X_4_ X_6_ = interaction of tween 80 and manganese sulphate tetrahydrate.

The R2 coefficient of the FFD model was 0.95, inferring that the model explained 95% of the data variability. The statistical significance of the model was confirmed by F-test. The F-value of 33.99 suggested that the model was significant and there was only a 0.01% chance that a model F-value this large could occur due to noise. Adequate precision measures the signal to noise ratio and generally a ration greater than 4 is desirable. The precision ratio of 17.36 indicated an adequate signal and this model could be used to navigate the design space.

### Concentration refinement of significant ionic minerals and non-ionic surfactant by CCD

CCD of RSM further refined the concentration of ionic minerals (sodium acetate and manganese sulphate) and non-ionic surfactant (Tween 80) identified by FFD. The concentrations tested for sodium acetate, Tween 80 and manganese sulphate were 0–10 g/L, 0–2 g/L and 0–0.08 g/L, respectively. The responses, together with the coded and actual values of selected variables are presented in Table 6. The highest experimental value for antimicrobial activity was 1280 MAU/mL. The biomass of *L. plantarum* RS5 was in the range of 1.95 g/L to 3.51 g/L. Interestingly, the observation of CCD was also noted in FFD, whereby significantly higher lactate and lower pH were detected simultaneously with higher antimicrobial activity and biomass, indicating both antimicrobial activity and lactate production by *L. plantarum* RS5 were growth associated.

**Table 6:** CCD matrix with coded and real values of ionic minerals and non-ionic surfactant and their corresponding effects on the antimicrobial activity, lactate concentration and pH of postbiotic RS5, and biomass of *L. plantarum* RS5.

Run	Ionic minerals and non-ionic surfactant(g/L)*			Antimicrobial activity (MAU/mL)**	Biomass (g/L)**	Lactate (g/L)**	Final pH**
	X_1_	X_2_	X_3_				
1	2.03 (− 1)	0.41 (− 1)	0.02 (− 1)	480.00^d^ ± 0.00*	2.36^b^ ± 0.03	3.79^c^ ± 0.07	3.95^bc^ ± 0.05
2	2.03 (− 1)	1.59 (+ 1)	0.06 (+ 1)	560.00^cd^ ± 46.19	2.31^b^ ± 0.06	5.73^b^ ± 0.03	3.83^bcde^ ± 0.07
3	5.00 (0)	1.00 (0)	0.04 (0)	1280.00^a^ ± 0.00	3.46^a^ ± 0.03	7.07^a^ ± 0.03	3.72^cde^ ± 0.02
4	7.97 (+ 1)	1.59 (+ 1)	0.06 (+ 1)	1280.00^a^ ± 92.38	3.35^a^ ± 0.08	6.90^a^ ± 0.29	3.73^cde^ ± 0.01
5	5.00 (0)	0.00 (− α)	0.04 (0)	480.00^d^ ± 0.00	2.29^b^ ± 0.10	3.70^c^ ± 0.19	3.89^bcd^ ± 0.12
6	7.97 (+ 1)	1.59 (+ 1)	0.02 (− 1)	1120^a^ ± 0.00	3.22^a^ ± 0.01	7.03^a^ ± 0.03	3.70^de^ ± 0.01
7	5.00 (0)	1.00 (0)	0.04 (0)	1280.00^a^ ± 0.00	3.48^a^ ± 0.03	7.03^a^ ± 0.04	3.74^bcde^ ± 0.02
8	2.03 (− 1)	0.41 (− 1)	0.06 (+ 1)	480.00^d^ ± 0.00	2.42^b^ ± 0.07	3.84^c^ ± 0.08	3.97^b^ ± 0.08
9	7.97 (+ 1)	0.41 (− 1)	0.02 (− 1)	400.00^d^ ± 0.00	1.95^b^ ± 0.05	3.11^d^ ± 0.06	4.26^a^ ± 0.04
10	5.00 (0)	1.00 (0)	0.04 (0)	1280.00^a^ ± 92.38	3.47^a^ ± 0.03	7.00^a^ ± 0.05	3.70^de^ ± 0.01
11	5.00 (0)	1.00 (0)	0.04 (0)	1280.00^a^ ± 0.00	3.48^a^ ± 0.04	7.00^a^ ± 0.06	3.69^de^ ± 0.03
12	5.00 (0)	1.00 (0)	0.08 (+ α)	800.00^b^ ± 0.00	2.49^b^ ± 0.04	5.71^b^ ± 0.03	3.76^bcde^ ± 0.03
13	5.00 (0)	2.00 (+ α)	0.04 (0)	1120.00^a^ ± 0.00	3.23^a^ ± 0.03	6.99^a^ ± 0.08	3.69^de^ ± 0.02
14	0.00 (− α)	1.00 (0)	0.04 (0)	560.00^cd^ ± 0.00	2.24^b^ ± 0.04	5.56^b^ ± 0.03	3.87^bcde^ ± 0.02
15	2.03 (− 1)	1.59 (+ 1)	0.02 (− 1)	1120.00^a^ ± 0.00	3.20^a^ ± 0.09	6.92^b^ ± 0.05	3.68^de^ ± 0.04
16	5.00 (0)	1.00 (0)	0.00 (− α)	720.00^bc^ ± 46.19	2.43^b^ ± 0.05	5.70^b^ ± 0.01	3.77^bcde^ ± 0.03
17	5.00 (0)	1.00 (0)	0.04 (0)	1280.00^a^ ± 0.00	3.50^a^ ± 0.01	7.04^a^ ± 0.04	3.69^de^ ± 0.02
18	7.97 (+ 1)	0.41 (− 1)	0.06 (+ 1)	1120.00^a^ ± 0.00	3.44^a^ ± 0.30	6.91^b^ ± 0.14	3.67^de^ ± 0.03
19	10.00 (+ α)	1.00 (0)	0.04 (0)	1120.00^a^ ± 0.00	3.42^a^ ± 0.29	6.93^a^ ± 0.13	3.66^e^ ± 0.04
20	5.00 (0)	1.00 (0)	0.04 (0)	1280.00^a^ ± 92.38	3.51^a^ ± 0.06	6.99^a^ ± 0.10	3.71^de^ ± 0.01

*X_1_: Sodium acetate; X_2_: Tween 80; X_3_: manganese sulphate tetrahydrate. Coded values of medium components are indicated in parentheses.

**Values presented are mean ± standard error of the mean (SEM), n = 3. Mean ± SEM within the same column that does not share a similar superscript are significantly different (p < 0.05).

By applying multiple regression analyses to the experimental data, the following quadratic equation 2 explains well the antimicrobial activity produced by *L. plantarum* RS5 under the effects of sodium acetate, Tween 80 and manganese sulphate:

2 $$\sqrt{\text{Y }}= +3.91+1.73 {\text{X}}_{1}+28.10 {\text{X}}_{2}+381.20 {\text{X}}_{3}-0.28 {{\text{X}}_{1}}^{2 }-7.80 {{\text{X}}_{2}}^{2 }-4951.68 {{\text{X}}_{3}}^{2 }+45.22 {\text{X}}_{1}{\text{X}}_{2}-185.24 {\text{X}}_{2}{\text{X}}_{3}$$

whereby Y = antimicrobial activity; X_1_ = sodium acetate; X_2_ = tween 80; X_3_ = manganese sulphate tetrahydrate.

The statistical significance of the quadratic equation was further confirmed by F-test. The ANOVA for the response surface quadratic model was shown in Table 7. The F-value, of 471.97 and the probability value at P > F was < 0.0001, implying the model was highly significant. There was just 0.01% chance that a “Model F-value” this large could occur because of noise. The coefficient that estimated the quadratic equation, along with the corresponding P values was indicated in the ANOVA analyses. The linear coefficients (X_1_, X_2_), all quadratic coefficients, and two interaction coefficients, i.e. X_1_X_3_ and X_2_X_3_, were highly significant (p < 0.01). The coefficient of R^2^ value evaluated the goodness of the model. The R^2^ value closed to 1.0 reflected the model’s strength to predict the response more effectively. Meanwhile, the adequate precision of 54.77 showed that this polynomial quadratic model had an adequate signal and could be used to navigate the design space.

**Table 7:** ANOVA analyses for CCD models of ionic minerals and non-ionic surfactant on the antimicrobial activity of postbiotic RS5 produced by *L. plantarum* RS5.

Ionic minerals and non-ionic surfactant*	Sum of squares	df	Mean square	F value	Prob > F**
Model	679.99	9	75.55	471.97	< 0.0001
X_1_	107.11	1	107.11	669.10	< 0.0001
X_2_	172.43	1	172.43	1077.16	< 0.0001
X_3_	5.19	1	5.19	32.40	0.0002
X_1_^2^	86.16	1	86.16	538.23	< 0.0001
X_2_^2^	109.42	1	109.42	683.51	< 0.0001
X_3_^2^	113.07	1	113.07	706.34	< 0.0001
X_1_X_2_	0.76	1	0.76	4.74	0.05
X_1_X_3_	81.79	1	81.79	510.91	< 0.0001
X_2_X_3_	54.90	1	54.90	342.97	< 0.0001
R-squared	0.9977				
Adjusted R-squared	0.9955				
Adequate precision	54.77				

*X_1_: sodium acetate; X_2_: Tween 80; X_3_: manganese sulphate tetrahydrate.

**Value of “Prob > F” less than 0.05 indicates that the model terms are significant.

**Figure 4:**
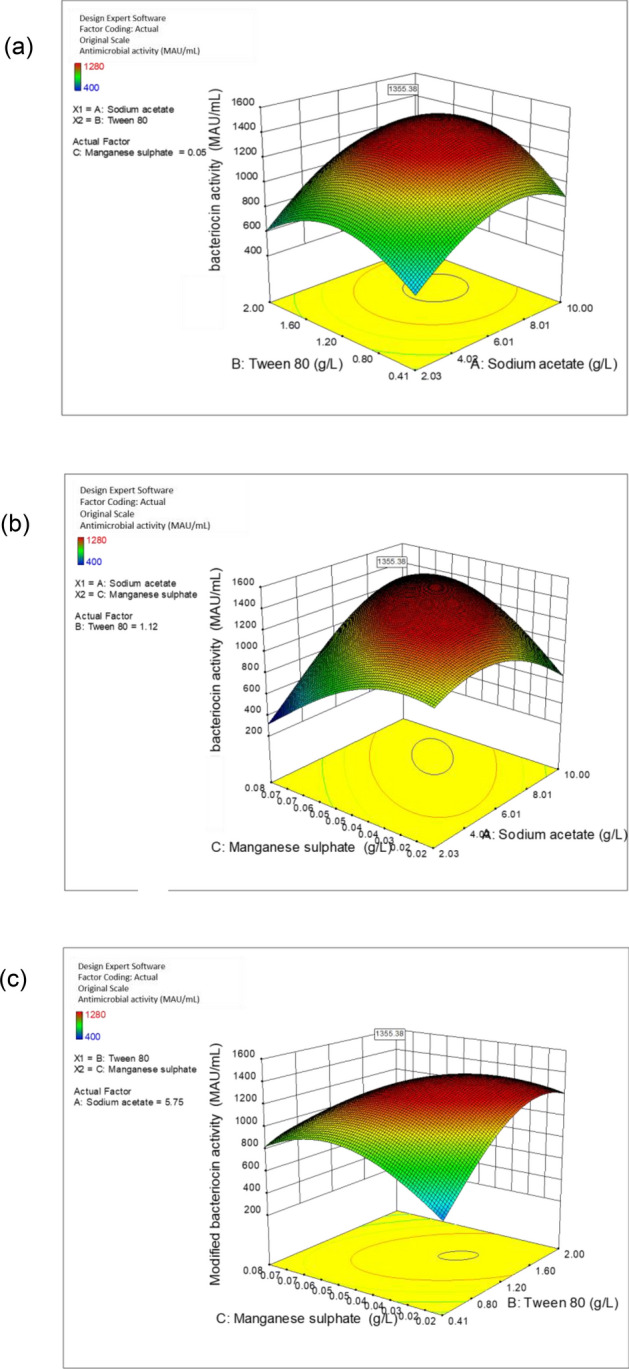
Response surface plots of antimicrobial activity of postbiotic RS5 produced by *L. plantarum* RS5 as a function of: (**a**) sodium acetate and Tween 80, (**b**) sodium acetate and manganese sulphate tetrahydrate, (**c**) Tween 80 and manganese sulphate tetrahydrate.

The three-dimensional response surface plots of Fig. 4 illustrate the interaction effects of sodium acetate, Tween 80 and manganese sulphate on the antimicrobial activity of postbiotic RS5. Higher antimicrobial activity was achieved when sodium acetate, Tween 80 and manganese sulphate were supplemented in growth medium (Table 6). The optimum values of sodium acetate, Tween 80 and manganese sulphate were determined from the response surface plots. The quadratic model predicted the antimicrobial activity of 1355 MAU/mL could be achieved by using the CCD refined medium. Subsequently, the predicted antimicrobial activity was validated through experiment and 1333.33 MAU/mL was attained, which was comparable to the predicted antimicrobial activity of 1355 MAU/mL. A biomass of 3.48 g/L was achieved when *L. plantarum* RS5 was grown in CCD refined medium. The CCD refined medium consisted of glucose 20 g/L, yeast extract 27.84 g/L, sodium acetate 5.75 g/L, Tween 80 1.12 g/L, and manganese sulphate tetrahydrate 0.05 g/L. Tables 1 and 2 of supplementary results show the cost of the CCD refined growth medium reduced by 85% as compared to the control MRS medium.

## Discussion

### Determination of antimicrobial activity

Our previous studies have demonstrated that the postbiotic RS5 exhibited broad inhibitory activity against *P. acidilactici*, *L. monocytogenes*, *S. enterica, E. coli* and Vancomycin resistant enterococci^18,19^. LAB species that are tolerant to acidic condition have been commonly employed to determine the antimicrobial activity attributed to bacteriocin molecules that are present in crude postbiotic preparations. Therefore, *P. acidilactici* ATCC 4-46, a LAB that is tolerant to acidic condition, was employed as the indicator microorganism for the determination of antimicrobial activity in this study. The antimicrobial activity was expressed as modified arbitrary unit (MAU/mL) by considering the diameter of inhibition zone as described by Lim^5^ to determine the antimicrobial activity quantitatively.

### Optimum concentration of yeast extract nitrogen source

Previous research findings have reported that medium composition can have a pronounced effect on the production of bacteriocin and the growth of LAB producer cells^28–32^. Therefore, we adopted a combination of conventional and statistical-based approaches to develop a refined medium to enhance the antimicrobial activity of postbiotic RS5. Yeast extract has been reported to have a strong effect on bacteriocin production by *Lactiplantibacillus* sp.^30,34,46^. Higher bacteriocin production could be due to inactivation of a bacteriocin synthesis inhibitor^39,40^. Moreover, yeast extract contains more growth factors and a relatively more free amino acids and short peptides than many other nutrient sources. These compounds could stimulate bacteriocin production by LAB as compared to other protein hydrolysates^30,34^. The results obtained in this study also indicated that the antimicrobial activity of postbiotic RS5 was significantly affected by the concentration of yeast extract supplemented in the growth medium.

The biphasic exponential production of antimicrobial activity (Fig. 1a) was observed in growth media containing 27.84 g/L (200.18 mM N) and 36.20 g/L (260.28 mM N) of yeast extract, respectively. Moghadam et al.^47^ demonstrated that *L. plantarum* RS5 harboured both plnEF and plnW structural genes, which encode Plantaricin W lantibiotic (class 1) bacteriocin and Plantaricin EF non-lantibiotic (class 2) bacteriocin molecules^48^. The biphasic production of antimicrobial activity could be attributed to the production of Plantaricin W and Plantaricin EF by *L. plantarum* RS5. However, further study should be performed to confirm the type of bacteriocins that could account for the biphasic antimicrobial activities produced in postbiotic RS5 under these conditions.

The antimicrobial activity was enhanced 43% when the yeast extract concentration was increased from 11.89 to 36.20 g/L. Todorov et al.^49^ reported a twofold increase in bacteriocin activity (3200 AU/mL) produced by *L. sakei*, when 20 g/L of yeast extract was supplemented. Similarly, the production of bacteriocin-like activity by *Enterococcus durans* E204 was increased from 320 to 640 AU/mL, when the yeast extract concentration was increased from 0.5 to 2.0%^50^. However, a twofold reduction of antimicrobial activity of postbiotic RS5 was noted when yeast extract was increased to 44.55 g/L, indicating that higher concentration of yeast extract could have induced substrate inhibition on bacteriocin synthesis when excessive nitrogen source was supplemented in the growth medium. The substrate inhibition observation was also reported for bacteriocin activity of *L. rhamnosus*^50^ and *L. brevis* DF01^34^, respectively.

*Lactiplantibacillus plantarum* RS5 produced different levels of antimicrobial activities and biomass when different carbon sources were supplemented in the growth medium, demonstrating that it has different preference and ability to efficiently utilise different carbon sources. This could be related to a wide range of hydrolytic enzymes required for the metabolism of different carbohydrates. Carbohydrate catabolism provides energy to LAB via substrate-level phosphorylation for cell growth and metabolite production^52^. The extracellular hydrolytic enzyme activities have been reported recently for various LAB isolated from Malaysians foods, including *L. plantarum* RS5^43,44^. The diverse effects of different carbon sources on bacteriocin production by LAB have been reported previously^34,39,49–52^. The positive effect of glucose was reported for bacteriocin produced by *L. brevis* DF01^34^, *L. plantarum* I-UL4^46^ and *Streptococcus bovis*^52^. The results obtained in this study agreed with the observation reported by Todorov and Dicks^49^. Bacteriocin production was reduced by 50% and 87.5%, when *L. pentosus* was grown in medium containing sucrose and fructose, respectively. Xylose was reported to support bacteriocin production by *Lactococcus lactis*^39^. In contrast, fructose was a better carbon source for the bacteriocin production by *L. mesenteroides* E131 as compared to glucose^53^, suggesting that the carbon source requirement by LAB was varied and strain specific. Thus, it is important to test different carbon sources when aiming to enhance the antimicrobial activity produced by LAB.

The antimicrobial activities of postbiotic RS5 produced between 16 and 20 h of incubation using glucose-containing growth medium was significantly higher (p < 0.05) compared to when lactose was used as carbon source (Fig. 2a). Between 26 and 30 h of incubation, antimicrobial activity of 1120 MAU/ml was achieved by both lactose and glucose-containing growth medium. The delay in the achievement of the highest antimicrobial activity of 1120 MAU/ml when lactose was used as the sole carbon source could be due to the disaccharide nature of lactose as compared to the simple sugar of glucose. Glucose could be catabolised directly via the glycolytic pathway to supply energy for bacteriocin production. The disaccharide lactose has to be taken up by a specific permease to be hydrolysed by β-galactosidase to produce glucose and d-galactose monomers, which will be subsequently converted to glucose-6-phosphate via the Leloir pathway prior to catabolism by the glycolytic pathway.

The highest biomass of *L. plantarum* RS5 (3.03 g/L) was achieved with glucose-containing growth medium. Polak-Berecka et al.^54^ reported that the maximal biomass (5.50 g/L) of *L. rhamnosus* was achieved when it was cultivated in modified MRS medium supplemented with 13.40 g/L of glucose. Although *L. plantarum* RS5 produced comparable antimicrobial activity in lactose containing medium, the lowest specific growth rate (0.06 h^−1^) and biomass formation (1.73 g/L) were observed when lactose was supplemented as carbon source. A similar observation was also reported by Sanchez et al.^27^ for nisin Z production by *Lactococcus lactis* subsp. *lactis*. A higher amount of nisin Z and a lower amount of biomass were produced by *L. lactis* subsp. *lactis* when it was grown in a lactose-containing medium. The lactose could have induced the transcription of the nisZ structural gene^27^.

The effect of glucose concentration was also reported for bacteriocin production by different LAB^50^. Bacteriocin-like activity was detected at 160 AU/mL when *E. duran* E204 was cultured in a medium supplemented with 4.0% (w/v) glucose, which was 75% lower than the activity that was achieved with 3.0% (w/v) glucose. Similarly, bacteriocin production by *L. plantarum* LL441 was also significantly reduced when it was cultivated in the medium containing more than 3% (w/v) carbohydrates^55^. Homofermentative *Lactobacilli* have been reported to possess a phosphoenolpyruvate-sugar phosphotransferase system^56^. High glucose concentration has been shown to inhibit sugar uptake via phosphorylation of the heat-stable protein of the phosphoenolpyruvate-sugar phosphotransferase system^55^. The increased level of phosphorylated protein would eventually lead to a reduction in glucose intake and switching off the catabolite-sensitive operons^55^. Nonetheless, the synthesis of antimicrobial substances would be advantageous when the initial concentration of nutrient at an optimal level becomes limiting during the fermentation process. Under the limiting conditions, the bacteriocin would antagonise the potential competitors for exhausting nutrients such as glucose and yeast extract. This concept was proposed for antibiotic production^57^.

Both the highest antimicrobial activity and biomass were achieved in growth medium containing 20 g/L of glucose, inferring that the production of antimicrobial compounds by *L. plantarum* RS5 directly correlated to the growth, which agreed with the study reported by Trinetta et al.^58^. Moreover, the production of pediocin AcH^59^ and sakacin P^60^ by LAB were stimulated when the medium and cultivation condition favored the growth of bacteriocin producer cells. However, there are also some cases in which higher bacteriocin activity were achieved under sub-optimal growth conditions^61,62^. The optimal concentration of glucose (20 g/L) and yeast extract (27.84 g/L) contributed to an optimal C:N ratio of 3.33, which enhanced the antimicrobial activity of postbiotic RS5 significantly (p < 0.05). The optimal C:N ratio improved bacteriocin production by *Bifidobacterium thuringiensis*^63^ and *L. curvatus* L442^64^. A more than fourfold increase of bacteriocin production was reported when *B. thuringiensis* was cultured in medium with a C:N ratio of 9^63^. Similarly, nitrogen has been shown to be a limiting factor for bacteriocin synthesis by *L. curvatus* L442, and the optimal ratio of nitrogen and carbon sources has been demonstrated to substantially enhance bacteriocin activity^64^.

### Statistical refinement of ionic minerals and non-ionic surfactant

Tween 80 has been shown to be a crucial factor for bacteriocin production^30,45^. The inclusion of Tween 80 in the growth medium improved the production of bacteriocin ST194BZ by more than 50%^65^. Tween 80 plays a significant role as non-ionic surfactant that facilitates bacteriocin secretion by altering membrane fluidity^30^. The ionic minerals are essential components for biological production processes^66,67^. Different effects of manganese sulphate were reported for bacteriocin production by different LAB isolates. The supplementation of manganese sulphate in the growth medium improved bacteriocin production by *L. pentosus*^30^ and *L. plantarum*^55^. However, manganese sulphate exerted no effect on bacteriocin production by *L. brevis* OG1^68^ and an inhibitory effect on salivaricin production by *L. salivarius* CRL 1328^66^. The inhibitory effect of manganese sulphate on salivaricin production could be attributed to the growth stimulation of *L. salivarius*, whereby the available nutrients were used for biomass production. In the present study, manganese sulphate had a significant stimulatory effect on the antimicrobial activity of postbiotic RS5. In contrast, the other ionic minerals: Na^+^, K^+^ and Mg^2+^ did not show any significant linear effect on antimicrobial activity of postbiotic RS5, which was contradictory to the results reported for micrococcin production, whereby significant effects of Mg^2+^ and K^+^ were noted for micrococcin production by *Micrococcus* sp. Moreover, *Micrococcus* sp. produced a 32-fold increase of micrococcin activity, when 2.5% K_2_HP0_4_ and 0.5% MgS0_4_.7H_2_0 were supplemented in the optimised medium, as compared to the control MRS medium^67^. However, MgSO_4_ has been demonstrated to exert a positive effect and K_2_HPO_4_ exerted an inhibitory effect on the bacteriocin production by *L. salivarius*^66^.

Citrate and phosphate ions did not enhance the antimicrobial activity of postbiotic RS5. This observation has further supported the notion that LAB, like *L. plantarum,* preferred an acidic environment to produce defensive molecules^57,69^. Phosphate control is a well-known regulatory mechanism to produce an inhibitory substance^70,71^. However, not all inhibitory substances were favourably produced under the presence of phosphate. The beneficial effect of phosphate was reported for nisin production by *L. lactis* strain^39,72^. But the negative effect of phosphate was demonstrated for both antimicrobial activities of postbiotic RS5 and salivaricin production^66^. The negative effect of phosphates could be due to a production inhibition mechanism, release or action of synthetases, inducers, precursors and other molecules, which were involved in the synthesis of inhibitory substance^70,71^.

The linear effect of Tween 80 (X4) and manganese sulphate (X6), the interaction effect between sodium acetate (X1) and Tween 80 (X4), and between Tween 80 (X4) and manganese sulphate (X6) strongly influenced the production of antimicrobial activity by *L. plantarum* RS5. The positive interaction between manganese sulphate and sodium acetate on antimicrobial activity of postbiotic RS5 agreed with a previous report, whereby salivaricin production reached the highest level when Mn^2+^ and Na^+^ were simultaneously added to the growth medium^64^. However, the presence of acetate, sulphate, citrate, and phosphate anions in growth medium exerted different effects on the antimicrobial activity of postbiotic RS5, whereby significant positive effects of acetate and sulphate were observed in this study. Similarly, the inclusion of acetate ions in the growth medium has been reported to increase the bacteriocin production by *L. salivarius*, attributed to the buffering capability of acetate ions to maintain the pH of the growth medium below 7^66^. In the current study, the concurrent addition of acetate and sulphate ions (0.05 g/L) in the growth medium increased the antimicrobial activity of postbiotic RS5, suggesting that the production of antimicrobial activity was favourable when mild acidic condition was maintained during the growth of *L. plantarum* RS5.

The lowest pH of 3.66 was noted in postbiotic RS5, demonstrating antimicrobial activity of 1120 MAU/mL (Run 19), did not correspond to the highest antimicrobial activity of 1280 MAU/mL and the highest concentration of lactate of 7.07 g/L of Run 3. Nonetheless, significantly higher (p < 0.05) biomass of *L. plantarum* RS5 (3.46–3.51 g/L and) and lactate concentration (6.90–7.07 g/L) were observed in postbiotic RS5 exhibiting the highest antimicrobial activity of 1280 MAU/mL, implying that the antimicrobial activity and lactate production by *L. plantarum* RS5 were growth associated. A similar observation was also reported by Chaline et al.^73^, whereby maximum lactic acid production was found in the growth medium that produced the maximum biomass of *L. plantarum* BL011.

## Conclusions

The combination of conventional and statistical-based optimisation techniques (FFD and CCD of RSM) was demonstrated to be a useful tool to develop a refined growth medium to significantly enhance the antimicrobial activity of postbiotic RS5. The defined growth medium that consisted of 20 g/L glucose, 27.84 g/L yeast extract, 5.75 g/L sodium acetate, 1.12 g/L Tween 80 and 0.05 g/L manganese sulphate exerted a significant impact on *L. plantarum* RS5 to produce an enhanced antimicrobial activity of 1333.33 MAU/mL. The antimicrobial activity was enhanced 108% compared to 640 MAU/mL antimicrobial activity that was attained using control MRS medium. The highest biomass concentration of 3.48 g/L was also obtained when *L. plantarum* RS5 was cultivated in the refined growth medium. The cost of the refined growth medium was substantially reduced, by 85%, compared to the control MRS medium. The results obtained in the current study have shown that significant enhancement of the antimicrobial activity of postbiotic RS5 was successfully achieved by refining the growth medium composition via a combination of conventional and statistical-based approaches. The improvements of growth medium have substantially lowered the production cost of the postbiotic RS5 and make it more feasible for the postbiotic RS5 to be used as a feed supplement in various animal production industries.

## Materials and methods

### Microorganisms

*Lactiplantibacillus plantarum* RS5 was used to produce postbiotic RS5. *P. acidilactici* ATCC 4-46 was employed as an indicator microorganism for the determination of antimicrobial activity^5^. *L. plantarum* RS5 was previously isolated from Malaysian steamed fish^4^. Both microorganisms were obtained from the Laboratory of Industrial Biotechnology, Department of Bioprocess Technology, Faculty of Biotechnology and Biomolecular Sciences, Universiti Putra Malaysia, and were maintained in MRS [Merck, Darmstadt, Germany] medium at − 20 °C supplemented with 20% [v/v] glycerol [Merck, Darmstadt, Germany]. They were revived in MRS medium (Merck, Darmstadt, Germany) according to the method of Foo et al.^74^.

### Preparation of postbiotic RS5

The postbiotic produced by *L. plantarum* RS5 was designated as postbiotic RS5 in this study. The preparation of postbiotic RS5 was performed according to the method of Ooi et al.^46^. The postbiotic RS5 was collected by centrifugation (10,000×*g* for 15 min at 4 °C) and kept at 4 °C for the determination of antimicrobial activity^5,75^.

### Determination of antimicrobial activity

The antimicrobial activity of postbiotic RS5 was determined using a modified Agar Well Diffusion Assay^5,75^, without neutralising the pH of the postbiotic RS5. A twofold serial dilution (2^0^ to 2^–5^) was conducted to dilute postbiotic RS5 using sterile 0.85% (w/v) NaCl solution. Diluted postbiotic RS5 (20 µL) was then allowed to diffuse around a well (5.0 mm in diameter) pre-punched in MRS agar medium, followed by overlaying with 3 mL of soft agar inoculated with 1% (v/v) *P. acidilactici* 4-46 (OD_600nm_ was adjusted to 1.0) before incubating at 30 °C for 24 h. A clear inhibition zone with a diameter of more than 1 cm (including 0.5 cm diameter of the well) was considered a positive antimicrobial activity. The antimicrobial activity was expressed as modified arbitrary unit (MAU/mL) using the following equation 3^3^:

3 $$\begin{aligned}& {\text{Modified arbitrary unit }}\left[ {{\text{MAU}}/{\text{mL}}} \right] \, \hfill \\ & = \left[ {\frac{{{\text{Reciprocal of the highest dilution factor yielded clear zone }}\left[ {{\text{AU}}} \right]}}{{{\text{Volume of postbiotic generated positive antimicrobial activity }}\left[ {{\text{mL}}} \right]}}} \right] \hfill \\ & \quad \times {\text{Clear zone diameter }}\left[ {{\text{cm}}} \right] \hfill \\ \end{aligned}$$

### Determination of microbial biomass

Microbial biomass was determined according to the method described by Li and de Orduna^76^. A volume of 5 mL of the cultured broth was filtered through a pre-dried and pre-weighed 0.45 µm cellulose acetate filter paper (Sartorius Stedim Biotech, Germany). The filter paper was then dried overnight at 105 °C. The dried filter paper was then transferred to a desiccator for 4 h to allow the filter paper to cool down to room temperature. The difference between the final and initial weight of filter paper was taken as the weight of microbial biomass in the 5 mL of cultured broth. The microbial biomass was expressed as gram of dry weight per litre (g/L).

### Determination of lactate concentration and pH

Lactate concentration of postbiotic RS5 was determined using the Pico Trace Glucose Analyser (Trace Analytics GmbH, Germany) according to the manufacturer’s instruction and the pH of the postbiotic RS5 was determined using a pH meter (Mettler Toledo, US).

### Conventional refinement of antimicrobial activity of postbiotic RS5

The composition of MRS (Merck, Darmstadt, Germany) broth medium (Table 8) was used as a reference to refine the growth medium of *L. plantarum* RS5. The concentration of glucose and yeast extract were optimised by using a conventional approach^30,52^, whereas the ionic minerals and Tween-80 were optimised by using statistical approaches^34,35^. A volume of 1% (v/v) active inoculum (OD _600 nm_ = 1.0) was inoculated into 15 mL of growth medium and incubated at 30 °C under static condition. Each optimisation experiment was performed in triplicate.

**Table 8:** Composition of de Man,Rogosa and Sharpe (MRS) medium.

Composition	Concentration (g/L)
d (+) glucose	20.00
Meat extract	8.00
Peptone from casein	10.00
Yeast extract	4.00
Sodium acetate	5.00
Di-ammonium hydrogen citrate	2.00
Di-potassium hydrogen phosphate	2.00
Tween 80	1.00
Magnesium sulphate heptahydrate	0.20
Manganese sulphate tetrahydrate	0.04

### Optimum concentration of yeast extract nitrogen source

The effect of different concentrations of N source was determined at 11.89, 27.84, 36.20 and 44.55 (g/L) of yeast extract (Ohly KAT, Germany). The concentration (g/L) of glucose was fixed at 20.0, sodium acetate at 5.0, diammonium hydrogen citrate at 2.0, dipotassium hydrogen phosphate at 2.0, Tween 80 at 1.0, magnesium sulphate heptahydrate at 0.2 and manganese sulphate tetrahydrate at 0.04, which followed the composition of MRS broth (Table 8; Merck, Darmstadt, Germany). Glucose, sodium acetate, diammonium hydrogen citrate, dipotassium hydrogen phosphate, Tween 80, magnesium sulphate heptahydrate and manganese sulphate tetrahydrate were purchased from Merck Company (Darmstadt, Germany). The experiment was performed in triplicate with 2 h sampling intervals.

### Optimum concentration of carbon source

The effects of glucose, lactose, fructose and sucrose (Merck, Darmstadt, Germany) as carbon sources were determined at 20 g/L with the optimum concentration (g/L) of yeast extract (verified in the previous experiment), sodium acetate at 5.0, diammonium hydrogen citrate at 2.0, dipotassium hydrogen phosphate at 2.0, Tween 80 at 1.0, magnesium sulphate heptahydrate at 0.2 and manganese sulphate tetrahydrate at 0.04, which followed the composition of MRS broth (Table 8; Merck, Darmstadt, Germany). Subsequently, the optimum concentration of the best carbon source was then determined at 0, 5, 10, 15, 20 and 40 (g/L). The experiment was performed in triplicate with 2 h sampling intervals.

### Statistical refinement of antimicrobial activity of postbiotic RS5

Statistical refinement of growth medium for the enhancement of the antimicrobial activity of postbiotic RS5 was performed using Design Expert statistical software version 6.0.10 (State-Ease Inc, Minneapolis, MN). The optimum concentration of carbon source and yeast extract (as described in previous sections) was used for both FFD and CCD experiments. Both FFD and CCD experiments were performed in triplicate, and sampling was performed at 36th hour of incubation.

#### Selection of significant ionic minerals and non-ionic surfactant by FFD

FFD was employed to determine the effect of ionic minerals (sodium acetate, diammonium hydrogen citrate, dipotassium hydrogen phosphate, magnesium sulphate heptahydrate, manganese sulphate tetrahydrate) and non-ionic surfactant, Tween 80, on the antimicrobial activity of postbiotic RS5. The effect of ionic minerals and non-ionic surfactant were examined at three different levels with a minimum (− 1), central (0) and maximum (+ 1) value of FFD, as shown in Table 4. FFD suggested 32 experimental runs. The response effect was analysed using analysis of variance (ANOVA), and the significance level of each component was determined at p-value < 0.05.

#### Concentration refinement of significant ionic minerals and non-ionic surfactant by CCD

The optimum concentration and the interaction effects of the three most significant ionic minerals and non-ionic surfactant (sodium acetate, Tween 80 and manganese sulphate) identified by FFD were then further refined using CCD. Each variable was evaluated at five levels (− α, − 1, 0, + 1, + α) as shown in Table 6. CCD suggested 20 experimental runs and made up to 2^3^ factorial design with eight cube points, augmented with six replications of the centre point (all variables at level 0) and the six-star points, i.e., points having one variable at an axial distance to the centre of ± α. The α value was 1.68 as calculated by the following equation 4:

4 $$\upalpha = \frac{{\left[ {\left[ {\sqrt {2^{n} + 2{\text{Xn}} + {\text{r}}} - 2^{{{n \mathord{\left/ {\vphantom {n 2}} \right. \kern-\nulldelimiterspace} 2}}} } \right]^{2} \times 2^{n} } \right]^{0.25} }}{\sqrt 2 }$$

The relationship among the variables was determined by fitting the second-order polynomial equation  5 for antimicrobial activity as the response obtained from the 20 experimental runs.

5 $$\begin{aligned} Y & = b_{0} + b_{1} x_{1} + b_{2} x_{2} + b_{3} x_{3} + b_{11} {x_{1}}^{2} + b_{22} {x_{2}}^{2} + b_{33} {x_{3}}^{2} \hfill \\ & \quad + b_{12} x_{1} x_{2} + b_{13} x_{1} x_{3} + b_{23} x_{2} x_{3} \hfill \\ \end{aligned}$$

whereby y is the predicted response variable, *b*_0_ is the intercept, *b*_1_, *b*_2,_ *b*_3_ are the linear coefficients, *b*_11_, *b*_22_ and *b*_33_ are the squared coefficients, *b*_12_, *b*_13_ and *b*_23_ are the interaction coefficients, *x*_1_, is the sodium acetate*, x*_2_ is the Tween 80, and *x*_3_ is the manganese sulphate. ANOVA was used to obtain the coefficients of the equation. The analyses were performed for the responses obtained from the 20 experimental runs.

### Estimation of kinetic parameters

The specific growth rate (µ), the yield coefficient for growth (Y_x/s_), the yield coefficient for product formation (Y_p/s_) and the productivity (Pr) were calculated as kinetic parameters according to the method reported by Sinclair and Kristiansen^77^.

### Statistical analysis

The results obtained in this study were analysed by one-way ANOVA using the General Linear Model procedure of Statistical Analysis System^78^. Duncan’s Multiple Range Test System was used to compare the significant difference between the mean at p < 0.05.

## Supplementary Information

Supplementary Information.
