# Supplementary material for: A refined medium to enhance the antimicrobial activity of postbiotic produced by Lactiplantibacillus plantarum RS5
Source: Sci Rep. 2021 Apr 7;11:7617. doi: 10.1038/s41598-021-87081-6 (PMC8027010; doi:10.1038/s41598-021-87081-6)
Supplement: Supplementary file 1 — Supplementary Information. [file 41598_2021_87081_MOESM1_ESM.docx]

**A Refined Medium to Enhance the Antimicrobial Activity of Postbiotic Produced by *Lactiplantibacillus plantarum* RS5**

**May Foong Ooi^1^, Hooi Ling Foo^1,2*^, Teck Chwen Loh^3,4*^, Rosfarizan Mohamad^1,5^,**

**Raha Abdul Rahim^2,6,7^, Arbakariya Ariff^1,2^**

^1^Department of Bioprocess Technology, Faculty of Biotechnology and Biomolecular Sciences, Universiti Putra Malaysia, 43400 UPM Serdang, Selangor, Malaysia.

^2^Institute of Bioscience, Universiti Putra Malaysia, 43400 UPM Serdang, Selangor, Malaysia.

^3^Department of Animal Science, Faculty of Agriculture, Universiti Putra Malaysia, 43400 UPM

Serdang, Selangor, Malaysia.

^4^Institute of Tropical Agriculture and Food Security, Universiti Putra Malaysia, 43400 UPM Serdang, Selangor, Malaysia.

^5^Institute of Tropical Forestry and Forest Products, Universiti Putra Malaysia, 43400 UPM Serdang, Selangor, Malaysia.

^6^Department of Cell and Molecular Biology, Faculty of Biotechnology and Biomolecular Sciences, Universiti Putra Malaysia.

^7^Office of Vice-Chancellor, Universiti Teknikal Malaysia Melaka, Jalan Hang Tuah Jaya, 76100 Durian Tunggal, Melaka, Malaysia.

Correspondence and requests for materials should be addressed to H.L.F.

(email: [hlfoo@upm.edu.my](mailto:hlfoo@upm.edu.my))

**Supplementary Results:**

**Comparison of the cost of commercially available de Man, Rogosa and Sharpe medium and refined medium for the enhancement of antimicrobial activity of postbiotic RS5 produced by *Lactiplantibacillus plantarum* RS5**

**Table 1: Cost of refined medium developed for the enhancement of antimicrobial activity of postbiotic RS5 produced by *Lactiplantibacillus plantarum* RS5**

| **Medium components** | | **Refined Medium components (g/L)** | **Cost of Refined Medium components (MYR)*** |
| --- | --- | --- | --- |
|  | Glucose | 20 | 0.83 |
|  | Yeast extract | 27.84 | 1.71 |
|  | Sodium acetate | 5.75 | 0.28 |
|  | Tween 80 | 1.12 | 0.36 |
|  | Manganese sulphate tetrahydrate | 0.05 | 0.02 |
|  | **Total cost (MYR)** |  | **3.20** |

Notes: *, the cost of refined medium components (MYR) per litre.

**Table 2: Comparison of the cost of commercially available de Man, Rogosa and Sharpe medium and refined medium for the enhancement of antimicrobial activity of postbiotic RS5 produced by *Lactiplantibacillus plantarum* RS5**

| Cost per liter (MYR)* | | Cost reduction (%) |
| --- | --- | --- |
| de Man, Rogosa and Sharpe medium | **Refined medium** |  |
| 22.60 | 3.20 | 85.6 |
